# Removal Behavior of Methylene Blue from Aqueous Solution by Tea Waste: Kinetics, Isotherms and Mechanism

**DOI:** 10.3390/ijerph15071321

**Published:** 2018-06-24

**Authors:** Li Liu, Shisuo Fan, Yang Li

**Affiliations:** 1 School of Physics and Electronic Engineering, Fuyang Normal University, Fuyang 236037, China; wenfan1986@163.com; 2 School of Resources and Environment, Anhui Agricultural University, Hefei 230036, China; 3 Guangzhou Key Laboratory of Environmental Catalysis and Pollution Control, School of Environmental Science and Engineering, Institute of Environmental Health and Pollution Control, Guangdong University of Technology, Guangzhou 510006, China; linziyi_ly@163.com

**Keywords:** tea waste, methylene blue, kinetics, isotherm, mechanism

## Abstract

Tea waste (biosorbent) was characterized by BET, SEM, FTIR, XPS, solid state ^13^C-NMR and applied to remove methylene blue (MB) from aqueous solution. The effect of different factors on MB removal, kinetics, isotherms and potential mechanism was investigated. The results showed that tea waste contains multiple organic functional groups. The optimum solid-to-liquid ratio for MB adsorption was 4.0 g·L^−1^ and the initial pH of the MB solution did not need to be adjusted to a certain value. The pseudo-second-order model could well fit the adsorption kinetic process. The adsorption process could be divided into two stages: a fast adsorption stage and a slow adsorption stage. The adsorption isotherm could be well described by Langmuir and Temkin isotherm models. The maximum adsorption amount could reach 113.1461 mg·g^−1^ based on Langmuir isotherm fitting. Desorption and reusability experiments showed that MB adsorption onto tea waste could be stable and could not cause secondary pollution. The interaction mechanism between tea waste and MB involved electrostatic attraction, hydrogen bond, ion exchange, π-π binding. The organic functional groups of tea waste played an important role during the MB removal process. Therefore, tea waste has the potential to act as an adsorbent to remove MB from aqueous solution.

## 1. Introduction

Dyeing industry wastewater can pose a significant risk to the eco-environment due to its color depth, high concentration, complex organic components, toxicity and poor bio-degradability characteristics [1]. Dyeing industry wastewater should be treated before discharge into natural water bodies. However, undesirable or illegal release of wastewater into the environment could also cause serious pollution. Therefore, the treatment of dyeing wastewater is crucial and indispensable.

The main approaches for dyeing industry wastewater disposal include chemical degradation, biological treatment, membrane separation, and adsorption [2]. Compared with other methods, the adsorption technique is currently widely applied to remove pollutants from dyeing wastewater, due to its many advantages, which include easier operation, low cost, and high efficiency [3]. The priority research focuses on the choice of the adsorbent and the investigation of mechanism. Biomass is an ideal material for adsorbent owing to its wide availability and low cost, especially biomass based on agricultural and forestry residues [4,5,6,7,8,9,10,11,12,13,14].

Tea waste is widely produced due to the development of tea industry. Resource utilization of tea waste is a major trend. Preparation of tea waste-based adsorbents had raised intereston account of its characteristics, involving surface structure, functional groups, wide sources, stable removal effect [15]. Tea waste had been prepared as an efficient adsorbent to remove pollutant from aqueous solutions [16]. Removal of dyeing industry wastewater by tea waste had been previously reported and those studies had been focused on adsorption kinetics and thermodynamics [17,18,19]. However, the adsorption mechanism and recycling utilization of tea waste for methylene blue removal has rarelyreceivedattention. Therefore, the purpose of this study was: (1) to characterize the structure and composition of tea waste; (2) to investigate the adsorption behavior of tea waste for methylene blue adsorption; (3) to explore the adsorption mechanism of tea waste for methylene blue.

## 2. Materials and Methods

### 2.1. Materials

Tea waste was collected from a tea factory located at the south of Anhui Province. First, the tea waste was pretreated to remove ash using deionized water. Then, pigments, theophylline and caffeine in the tea waste were eliminated using boiling water. Last, the tea waste was dried at 70 °C and crushed to pass a 100 mesh sieve. The tea waste was then stored for further experiments. The reagents methylene blue (MB), sodium hydroxide, and hydrochloric acid were analytical pure and was purchased from Sinopharm Chemical Reagent Co., Ltd. (Shanghai, China). The experimental water was deoxidized water (membrane treatment).

### 2.2. Batch Experiment

In each experiment, 20 mL of MB solution was placed in a 50 mL centrifuge tube. The initial concentration of MB solution was diluted to 100 mg·L^−1^ from stock solution (1000 mg·L^−1^). Different masses of tea waste were added to adjust the solid-to-liquid ratios in the range of 1–10 g·L^−1^ (dosage effect). Meanwhile, the pH value of MB was adjusted to value between 3.0–11.0 with 0.1 M HCl or 0.1 M NaOH solutions to investigate the effect of pH on adsorption. Then, the centrifuge tubes were shaken in a controller shaker (150 rpm) for 24 h at the 35 °C. After centrifugation, the supernatant was passed through a 0.45 μm filter membrane and was collected for measurement. The absorbance of the supernatant was determined by a spectrophotometer at a wavelength of 665 nm. The removal rate and adsorption amount was calculated using the following equations:

(1) $$R=\frac{\left(C_{0}-C_{e}\right)}{C_{0}}\times 100$$

(2) $$q_{e}=\frac{(C_{0}-C_{e})\times V}{m}$$

where *R* is the removal rate of MB (%). *C*_0_ and *C_e_* are respectively the MB concentrations at the initial time and equilibrium time (mg·L^−1^). *m* is the mass of tea waste (mg). *V* is the solution volume (mL). *q_e_* is the adsorbed amount of MB ontotea waste (mg·g^−1^).

### 2.3. Adsorption Kinetics

Based on the above experimental conditions, 100 mL of MB solution with the initial concentration of 100 mg·L^−1^ were placed in a 250 mL conical flask. The pH of the solution was not adjusted. Then, a certain mass of tea waste (0.4 g) was added into the solution and the mixture was agitated at 150 rpm in a shaker at 35 °C. Samples were collected at different timeintervals and analyzed by a spectrophotometer at a wavelength of 665 nm. Then, the amount of MB adsorbed onto he tea waste was calculated. Different kinetic models were applied to fit the adsorption process.

### 2.4. Adsorption Isotherm

Based on the above experimental conditions, 20 mL of MB solution of different initial concentrations (ranging between 100 and 500 mg·L^−1^) were placed in a 50 mL centrifuge tube. A certain mass of tea waste (0.08 g) was added into the solution in the centrifuge tube and the mixture was agitated at 150 rpm in a shaker at 35 °C for 24 h. The adsorption amount was calculated. Different isotherm models were used to describe the process.

### 2.5. Characterization

The textural properties of tea waste were analyzed with a surface area and porosity analyzer (Micromeritics TriStar II 3020, Norcross, GA, USA) at 77 K under a N_2_ atmosphere. The surface morphology of tea waste was characterized by scanning electron microscopy (HITACHI S-4800, Toyko, Japan).

The concentration of released metals (Ca^2+^, Na^+^, K^+^, Mg^2+^) from the tea waste in the supernatant of the equilibrium solution was analyzed by Inductively Coupled Plasma Optical Emission Spectroscopy (ICP-OES, 2100DV, PerkinElmer, Fremont, CA, USA). The corresponding release of Ca^2+^, Na^+^, K^+^, Mg^2+^ from the tea waste with deionzed water was used as control. The functional groups of tea waste were analyzed by Fourier Transform Infrared Spectroscopy (FTIR, Nicolette is50, Thermo Fisher Scientific, Waltham, MA, USA) using the KBr pellet technique. The valence of the C, O, N bound on the tea waste was determined by X-ray Photoelectron Spectrometer (XPS, ESCALAB 250Xi, Thermo Fisher Scientific, Waltham, MA, USA). Nuclear Magnetic Resonance spectra (CP-MAS ^13^C-NMR) of tea waste were determined at a frequency of 100 MHz using using an Avance III 400 spectrometer (Bruker, Basel, Switzerland). All experiments were run in a double resonance probe head using 4-mm sample rotors. ^13^C multiple ramped amplitude cross polarization/magic angle spinning (^13^C multi CP) NMR experiments were performed.

### 2.6. Desorption and Reusability Experiment

The solutions of MB (initial concentration: 300 mg·L^−1^) were stirred with tea waste (solid-to-liquid ratios: 1 g·L^−1^) for 10 h at 25 °C. The adsorbent was separated from the suspension by mixing with ethanol/acetic acid eluent (*v*/*v*: 9/1) for 10 h and ultrasonicated for 10 min. After ultrasonication the adsorbent was washed with water several times to remove the dye molecules and dried in an oven at 80 °C.

## 3. Results and Discussion

### 3.1. Characterization of Tea Waste

The surface area, pore volume and pore diameter of tea waste is 0.913 m^2^·g^−1^, 0.007 cm^3^·g^−1^, 2.611 nm, respectively. Compared with other adsorbents, such as activated carbon, the surface structure of tea waste was not well developed [20]. When tea waste acted as an adsorbent, its adsorption capacity was not limited by its surface structure but also depended on other mechanisms.

**Figure 1:**
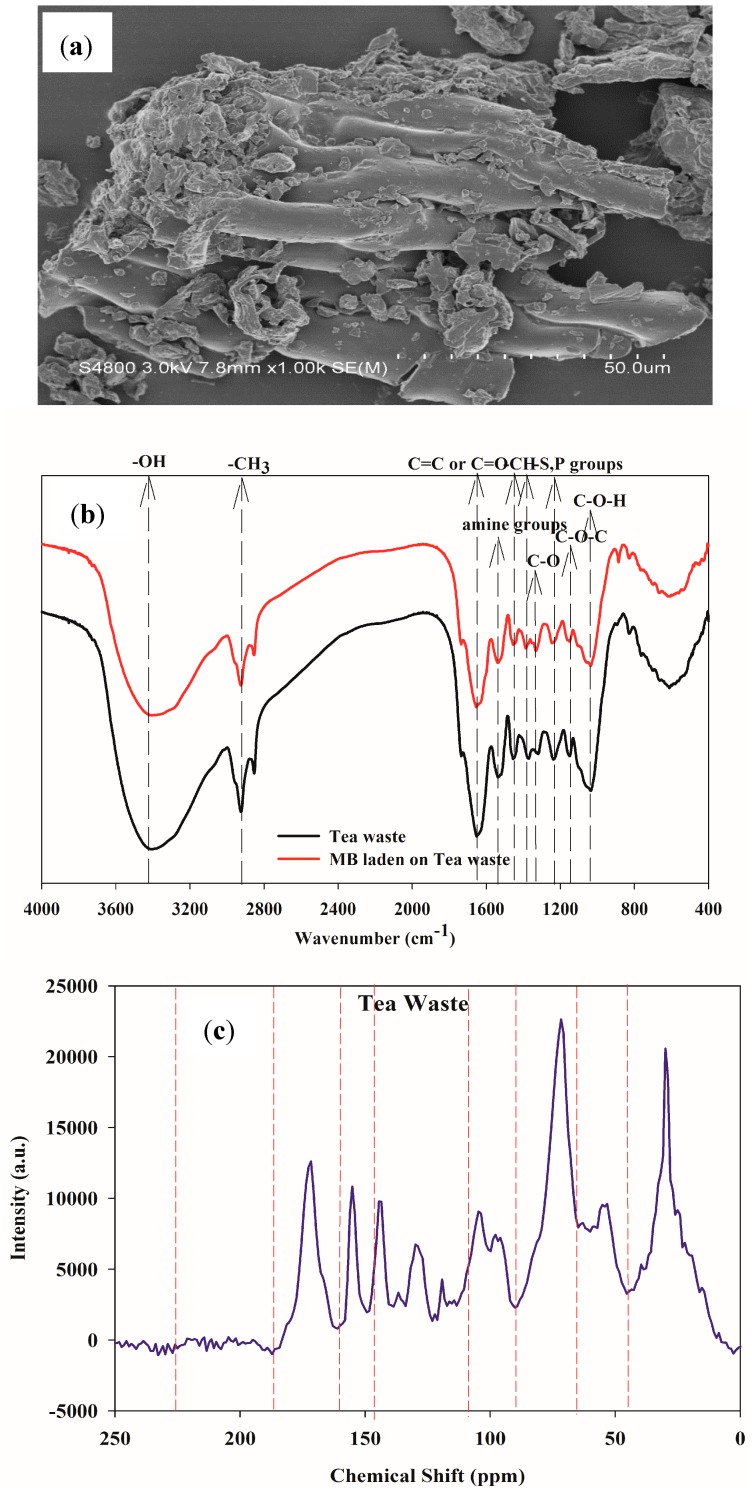
SEM photo (**a**), FTIR (**b**) and ^13^C-NMR (**c**) of tea waste.

A SEM micrograph of tea waste is shown in Figure 1a. Tea waste presented a stem structure due to its main components, including cellulose, and hemicellulose. Tea waste exhibits a heterogeneous or rough and porous (caves) surface structure which was favorable for the the biosorption of MB dye.

The FTIR of tea waste and MB loaded onto tea waste is presented in Figure 1b. The main functional groups of tea waste and MB laden on tea waste are summarized in Table 1. The functional groups of tea waste are as follows: –OH: 3416 cm^−1^, aliphatic –CH: 2924 cm^−1^ and 2852 cm^−1^, aromatic C=C and C=O: 1651 cm^−1^, secondary amine group: 1530 cm^−1^, N–H bending: 1455 cm^−1^, –CH_3_ bending: 1371 cm^−1^, C–O stretching: 1320 cm^−1^, –SO_3_ stretching/P=O: 1237 cm^−1^, C–O groups: 1150 cm^−1^, C=O groups: 1036 cm^−1^ [21,22,23,24].

When MB was adsorbed onto tea waste, part peaks of functional groups shifted, such as 3416 cm^−1^→3406 cm^−1^, 1651 cm^−1^→1644 cm^−1^, 1530 cm^−1^→1537 cm^−1^, 1371 cm^−1^→1385 cm^−1^, 1320 cm^−1^→1331 cm^−1^, 1237 cm^−1^→1244 cm^−1^. Therefore, the functional groups of –OH, –C=C or C=O, amine groups, –CH in tea waste may participate the interactions with MB, involving the mechanism of surface complex, hydrogen bonding, electrostatic attraction.

Solid state ^13^C-NMR spectroscopy has emerged as a useful tool to characterize different types of carbon. Figure 1c shows the ^13^C-NMR spectrumof tea waste. The relative proportion of different carbons in each chemical functional group for the tea waste is presented in Table 2. The relative proportions were integrated in the chemical shift (ppm) resonance intervals of 0–46, 46–65, 65–90, 90–108, 108–145, 145–160, 160–185, 185–225 ppm [25]. Obviously, O-alkyl and alkyl C with the chemical shift of 65–90 ppm and 0–46 ppm were the main C-containing functional groups in tea waste (sum: 46.37%). The O-alkyl and alkyl C refer to carboxyl and hydroxy functional groups. The results were in agreement with the FTIR analysis. Therefore, carbon and oxygen functional groups in tea waste may play a key role during the interaction with MB.

**Figure 2:**
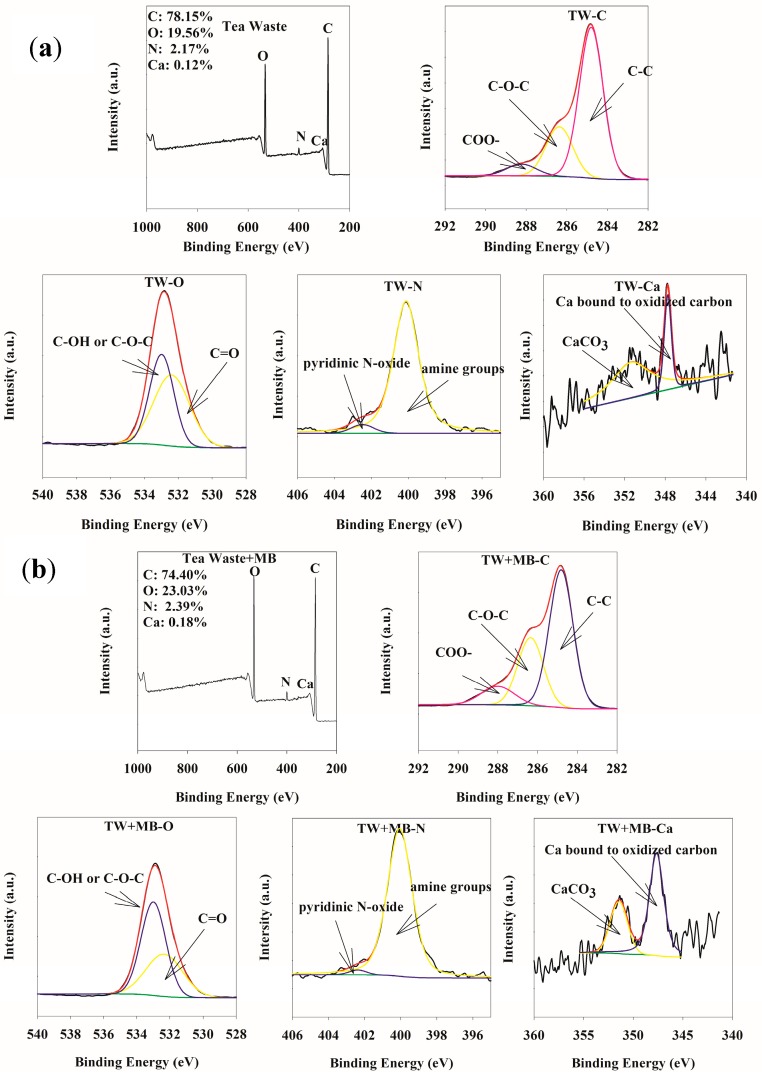
XPS spectra of tea waste (**a**) and MB loaded on tea waste (**b**).

The XPS analysis of tea waste is displayed in Figure 2. XPS could provide the information of surface element composition and speciation analysis. As shown in Figure 2a, C, O, N, Fe, Ca, K had been determined by XPS technique. However, Fe, K could not be detected in the tea waste due to the low content. C, O, N, Ca werethe main elements present in the tea waste. According to the XPS peak analysis, high-resolution spectra of C 1s of tea waste could be deconvoluted into three peaks: 284.8, 286.4 and 288.25 eV, which indicates presence of C=C/C–C, C–O–C and O–C=O [26,27]. The O 1s region contained two components, at 532.35 and 533.0 eV, corresponding to C=O and C–O–C or C–OH groups [28,29]. The peaks values are 400.10 and 402.5 eV, corresponding to protonated amine groups and N-H bonds, respectively [30,31,32]. Ca 2p_3/2_ peaks were at 347.4 and 351.20 eV and could be assigned to Ca bound to oxidized carbon, CaCO_3_ [33]. Therefore, abundant functional groups were present in the tea waste and lead to its adsorption properties.

When the MB was adsorbed onto tea waste, the XPS spectra of tea waste changed as shown in Figure 2b. Compared with raw tea waste, the main speciation of elements in the MB loaded onto tea waste slightly changed. The high-resolution spectra of C 1s of tea waste had been deconvoluted into three peaks: 284.8, 286.35 and 288 eV. The O 1s region contained two components, at 532.40 and 533.0 eV. The peaks values of N are 400.05 and 402.4 eV. Ca 2p3/2 peaks shifted to 347.4 and 351.20 eV. The variation of speciation of element indicated that these organic groups were involved in the interaction between tea waste and MB. The relative content of elements changed. Content of carbon decreased and the content of nitrogen, oxygen increased due to the adsorption of MB.

### 3.2. Adsorption Behavior

#### 3.2.1. Effect of S/L Ratios and pH on MB Adsorption

**Figure 3:**
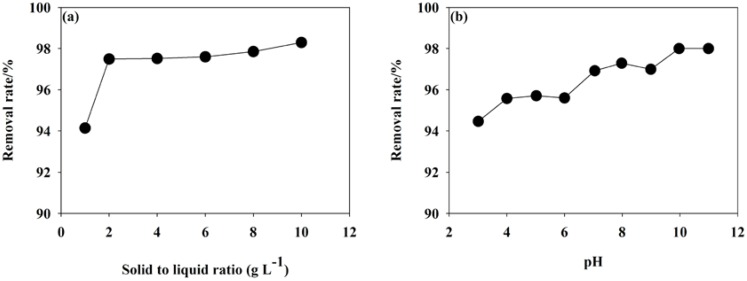
Effect of (**a**) S/L ratio and (**b**) pH on the removal rate of MB.

The effect of solid-to-liquid ratios and pH on MB removal by tea waste is shown in Figure 3. Solid-to-liquid ratio is an important factor influencing the MB removal effect. As shown in Figure 3a, as increase in the S/L ratio initially increased the removal rate of MB. The removal rate plateaued at a solid-to-liquid ratio of 4.0. When the S/L ratio was 4.0, the removal rate reached 98%. Thus, the optimum of S/L was 4.0. Higher S/L ratios indicated more mass of tea waste and could provide more adsorption sites for binding of MB and realized a higher removal effect. When the adsorption reached equilibrium, the removal rate was relative stable.

pH was a key factor during the adsorption process and affected the surface charge of the adsorbent, the degree of ionization, and the speciation of MB in the solution [34]. The effect of pH on MB removal effect is shown in Figure 3b. With increasing of pH the removal rate of pH tended to increase. When the pH was 3.0 and 11.0, the removal rate of MB was 94% and 98%, respectively. The influence of low pH to MB adsorption was that H^+^ could occupy the binding sites. This was not favorable for the adsorption of MB [35,36]. Further, MB possessed a positive surface charge and could be repulsed by H^+^ to prevent MB adsorption onto tea waste. With increasing pH the number of hydrogen ions in the solution reduced and the competitive effect, repulsive interaction weakened, which may lead to an increase in the removal rate. The MB removal rate became stable when the pH reached 8.0.

In this investigation, the removal rate was larger than 98%, when the pH of solution was not adjusted. Therefore, the pH was not adjusted in the subsequent experiments. Even at low pH value, the removal rate was still high (>94%) and meant other mechanism might be presented during the interaction between tea waste and MB.

#### 3.2.2. Adsorption Kinetics

Adsorption kinetics could be used to investigate the adsorption quantity changes over time and could provide reference for the engineering design process and gain an insight into the adsorption mechanism. In this study, varied nonlinear kinetics models were applied to fit the adsorption process [37,38,39].

Pseudo first-order model:

(3) $$q_{t}=q_{e}(1-e^{-k_{1}t})$$

where *q_e_* and *q_t_* are the amounts of MB adsorbed (mg·g^−1^) at equilibrium and at any time *t*. *k*_1_ is the pseudo first-order rate constant.

Pseudo-second-order model:

(4) $$q_{t}=\frac{k_{2}q_{e}^{2}t}{1+k_{2}q_{e}t}$$

where *k*_2_ is the pseudo-second-order rate constant.

Elovich model [40]:

(5) $$q_{t}=\frac{1}{\beta }\ln(\alpha \beta )+\frac{1}{\beta }\ln(t)$$

where *q_t_* (mg·g^−1^) is the same parameters as above mentioned. α (mg/(g·min)^−1^) is the initial adsorption rate, *β* (g·mg^−1^) is associate with the fraction of surface coverage and activation energy for chemisorptions.

Two-compartment model (TC model) [41,42]:

(6) $$q_{t}=q_{e}(1-(F_{\mathrm{fast}}e^{-k_{\mathrm{fast}}t}+F_{\mathrm{slow}}e^{-k_{\mathrm{slow}}t}))$$

F_fast_ and F_slow_ are the mass fractions and k_fast_ and k_slow_ are the first-order rate constants for transport into “fast” and “slow” compartment of the adsorbent, respectively.

**Figure 4:**
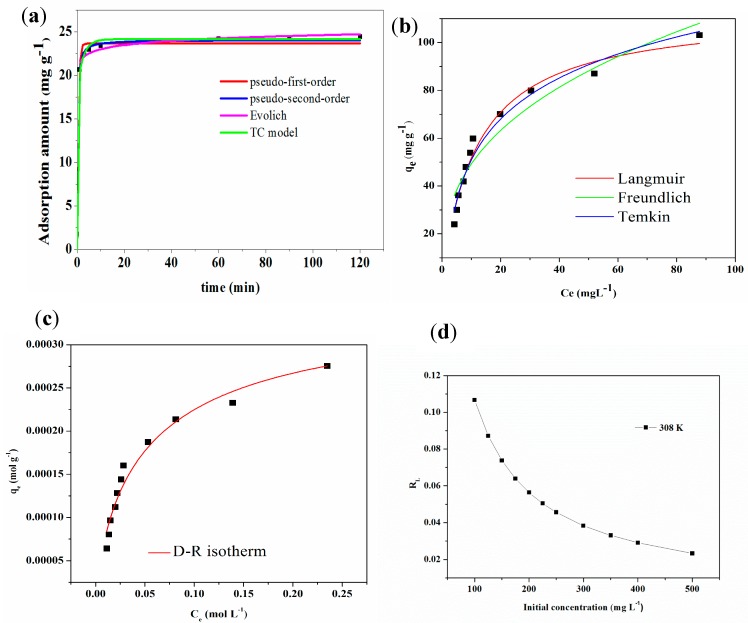
Kinetics (**a**), isotherm (**b**,**c**) curves and (**d**) R_L_ of MB adsorption by tea waste.

The nonlinear fitting curve of MB removed by tea waste is displayed in Figure 4a. The fitting parameters of different kinetics models are exhibited in Table 3. Compared with pseudo first-order, Evolich and TC models, the pseudo second-order model could better describe the adsorption process and the correlation coefficient was larger than 0.99. The pseudo-second order modelmeant that adsorption process was controlled by chemisorption which involved valency forces through sharing or exchange of electron between the solvent and the sorbate [43,44,45]. Because the pseudo-second order model contained the external liquid film diffusion, surface adsorption and intra-particle diffusion processes [46,47,48,49], this model could provide a more comprehensive and accurate description of the adsorption mechanism between tea waste and MB.

According to the TC models, the adsorption kinetics included an initial fast phase and final slow phase. The fast phase of adsorption could be finished within 5 min and removal rate was more than 90%. The slow phase of adsorption would be accomplished at the remaining time. Based on TC model, the mass fraction of “fast” and “slow” compartment is 78.99% and 21.01%, respectively. The F_fast_ value was greater than 0.5 and larger than those of F_slow_, indicating that fast adsorption stage was dominant during MB sorption process. Also, the first–order rate constant for transport “fast” compartment (k_fast_) was obvious lager than those of “slow” compartment (k_slow_).

#### 3.2.3. Adsorption Isotherm

Adsorption isotherm could be used to study the adsorption mechanism, predict the maximum adsorption capacity of adsorbent, estimate the affinity between adsorbent and adsorbate, optimize the design of adsorption system. In this study, varied nonlinear isotherm models were applied to fit the adsorption process [50], including Langmuir, Freundlich, Dubinin-Radushkevich, Temkin models [51,52,53,54]. The nonlinear fitting of the curve of MB remove by tea waste is displayed in Figure 4b,c. The fitting parameters of different isotherm models are listed in Table 3.

Langmuir model:

(7) $$q_{e}=\frac{Q_{0}b_{L}C_{e}}{1+b_{L}C_{e}}$$

where *C_e_* is the equilibrium concentration of MB (mg·L^−1^), *q_e_* is the amount of MB adsorbed per gram adsorbent at equilibrium (mg·g^−1^). *Q*_0_ is maximum adsorption capacity (mg·g^−1^), *b_L_* is the Langmuir isotherm constant (L·mg^−1^).

Langmuir could be fitted well to the adsorption process and the maximum adsorption capacity was 113.1461 mg·g^−1^. The potential of tea waste can be evaluated by comparing the adsorption capacity of MB onto various adsorbents (based on tea waste biomass) as shown in Table 4. The performance of the tea waste is clearly seen to be considerably effective for MB removal.

Dimensionless equilibrium constant, *R_L_*, referred to as the separate factor:

(8) $$R_{L}=\frac{1}{1+b_{L}C_{i}}$$

where *C_i_* is the initial concentration of MB (mg·L^−1^).

The value of *R_L_* lies between 0 and 1 for favorable adsorption, while *R_L_* > 1 means unfavorable adsorption, and *R_L_* = 1 represents linear adsorption while the adsorption process is irreversible if *R_L_* = 0 [55]. As shown in Figure 4d, *R_L_* is less than 1 in this investigation and demonstrates that adsorption process is effective and beneficial. Increased MB concentration could be beneficial for the adsorption behavior.

Freundlich model:

(9) $$q_{e}=K_{F}C_{e}{}^{1/n}$$

where *q_e_* is the amount of MB adsorbed per unit weight of adsorbent (mg·g^−1^), *C_e_* is the equilibrium concentration of solute in the bulk solution (mg·L^−1^). *K_F_* is the constant indicative of the relative adsorption capacity of the adsorbent (mg·g^−1^) and 1/*n* is the constant representing the intensity of the adsorption.

1/*n* in Freundlich equation could reflect the difficulty of adsorption behavior. Generally, when 1/*n* is less than 0.5, the adsorption process would be easily carried out. Adsorption process is difficult when the 1/*n* is larger than 0.5 [4,5]. In this research, the 1/*n* is less than 0.5 when MB was adsorbed onto tea waste, suggesting that adsorption behavior of methylene blue on tea waste easily performed.

Dubinin-Radushkevich model:

(10) $$q_{e}=Q_{m}e^{K\varepsilon^{2}}$$

(11) $$\varepsilon =RT\ln(1+\frac{1}{C_{e}})$$

(12) $$E=\frac{1}{\sqrt{2K}}$$

where *K* donates the coefficient related to the mean free energy of adsorption (mol^2^·kJ^2^), *Q_m_* is the maximum adsorption capacity (mg·g^−1^), and ε is the Polanyi potential, calculated by the equation. *R* is the gas constant (8.314 J·(mol·K)^−1^) and *T* is the absolute temperature (K). The mean adsorption energy *E* is calculated by the use of *K* values.

The mean adsorption energy provides important information related to the physical and chemical nature of the adsorption process. *E* is in the range of 8 kJ·mol^−1^, 8–16 kJ·mol^−1^, 16–40 kJ·mol^−1^, it corresponds to the physical interaction, ion exchange, chemical interaction, respectively [23]. In this study, the *E* of MB adsorbed on tea waste is 6.956 kJ·mol^−1^, indicating that physical interactions could play an important role during the adsorption process.

Temkin model:

(13) $$q_{e}=\frac{RT}{b_{T}}\ln(A_{T}C_{e})$$

where *A_T_*, Temkin isotherm equilibrium binding constant (L·mg^−1^) and *b_T_*, Temkin isotherm constant (J·mol^−1^).

The Temkin isotherm model assumes that the heat of adsorption of all the molecules in the layer decreases linearly with coverage due to adsorbent–adsorbate interactions and mainly describes the chemisoprtion process which dominated through electrostatic adsorption [4,5]. In this investigation, the correlation coefficient of Temkin model was larger than 0.95, revealing that electrostatic interaction was an important mechanism between tea waste and methylene blue.

### 3.3. Desorption and Reusability Performance

Desorption and reusability properties are important indicators to assess the stability of adsorbent. The reusability of tea waste towards adsorption of methylene blue dye molecules was studied for three cycles. The adsorption of MB (initial concentration: 300 mg·L^−1^) was stirred with the tea waste (solid-to-liquid ratios: 1 g·L^−1^) for 10 h at 25 °C. The adsorbent was separated from the suspension by mixing with ethanol/acetic acid eluent (*v*/*v*: 9/1) for 10 h and ultrasonicated for 10 min. After ultrasonication, the adsorbent was washed with water for several times to remove the dye molecules and dried in an oven at 80 °C.

**Figure 5:**
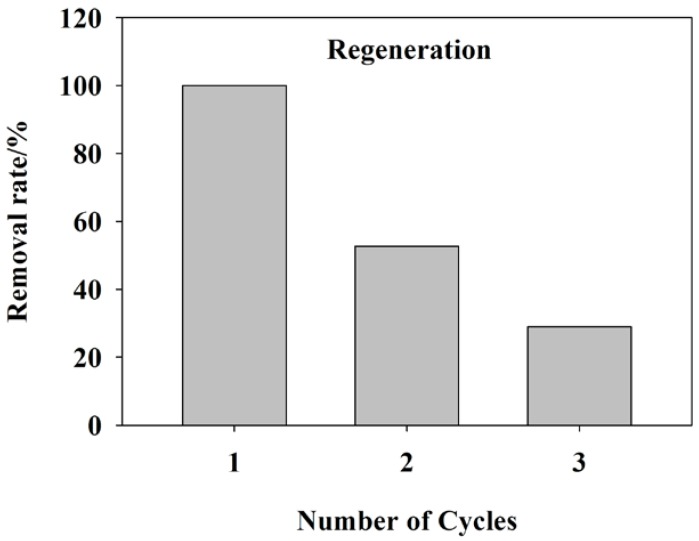
Desorption and reusability study for adsorption of methyl blue onto tea waste.

The removal rate of MB by tea waste is shown in Figure 5. The removal rate dropped to about 30% after three cycles. The removal rate of desorbed-tea waste decreased due to the change of superficial structure of tea waste and loss of the binding site in tea waste. In previous research, decrease of removal rate could be attributed the solubilized some parts of tea waste, changed superficial structures of tea waste and subsequently led to loss or blockage of adsorption sites [56,57]. Meanwhile, the desorption experiment indicated that tea waste had the potential to be adsorbent for MB removal and could not cause secondary pollution.

### 3.4. Recommended Adsorption Mechanism

The results of different factors on MB adsorption, adsorption kinetics and isotherm are summarized. The above analysis shows that the adsorption of methylene blue onto tea waste was a complicated process, involving multiple steps and various interactions.

Based on the results of TC models, the adsorption process could be divided into fast stage and slow stage. The fast adsorption stage could be finished in 5 min and the removal rate reached 79%. The slow adsorption stage could be accomplished by the remaining time and the removal rate is 21%. The fast adsorption stage could be explained by the following reason: the electrostatic ion exchange between MB and organic functional groups derived from tea waste is commonly considered to be fast and may reach higher removal rate in few minutes. Meanwhile, hydrogen bond or π-π stacking interaction between MB and tea waste may also exist during the adsorption process. This result was consistent with the analysis of the material by FTIR and XPS, which showed that organic functional groups obvious shifted after MB adsorbed onto tea waste. Therefore, organic functional groups of tea waste played an important role during the MB adsorption.

The pseudo second-order model could fit the kinetics data of MB adsorption onto tea waste, suggesting that chemisorptioninteractions play a dominant role during the adsorption process. Meanwhile, the interaction between tea waste and MB involved physical mechanism on account of E derived from D-R model. According to the results of pH effect, electrostatic interaction existed between tea waste and methylene blue. However, the electrostatic interaction was not the sole mechanism.

The MB molecular could occur with tea waste through surface complextion and H^+^, Ca^2+^, Mg^2+^ ions could be released into the solution. The decrease of pH from and cation detected in the solution could confirm the mechanism. When the adsorption process reached equilibrium, the pH of methylene blue solution from 7.98, 7.05, 6.00 decreased to 6.64, 5.98, 5.20 based on the results of pH measurement. Cation exchange was an important mechanism for adsorption of MB onto the tea waste surface. According to previous research, cation exchange is a key mechanism for MB adsorption onto adsorbent [58]. The net amount of released cations (mequiv·g^−1^) was calculated and is presented in Table 5. As seen in Table 5, net amount of released cations increased after MB adsorption and indicated that more cations were released to supernatant, especially the Ca^2+^ and Na^+^ ions which more participates the ion exchange process.

**Figure 6:**
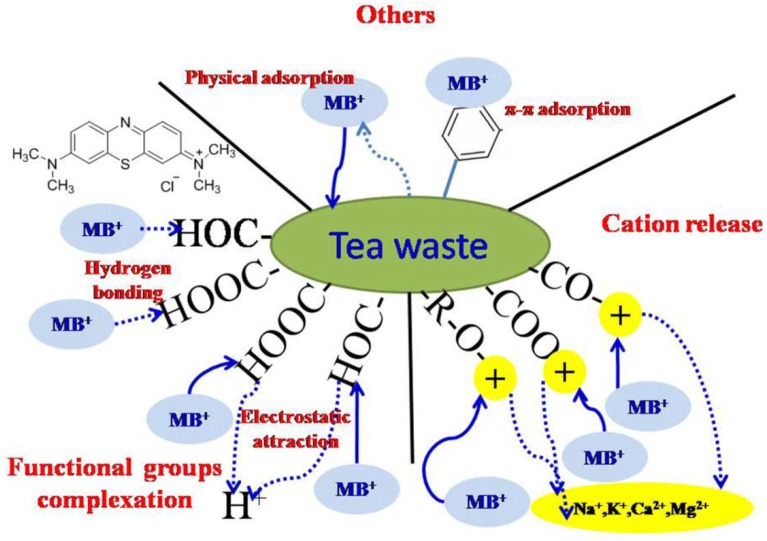
Mechanism diagram between tea waste and methylene blue.

The two benzene rings contained in methylene blue easily form π-π stacking interaction with the aromatic rings in the tea waste [59]. In short, the interaction between tea waste and MB referred to various mechanisms, including electrostatic interaction, hydrogen bond, ion exchange, π-π binding. Other research also found the similar mechanism [59,60,61,62,63,64]. The recommended mechanism diagram is displayed in Figure 6.

### 3.5. Environmental Significance of This Work

Large amounts of tea waste were produced in our province. Tea waste could be collected from tea factories or surrounding residents. Tea waste was washed with water to remove impurities. Then, tea waste could be dried through solar energy drying. The dried tea waste was crushed using a grinder. Pulverized tea waste could be sent to dyeing industries in local area and could be acted as an adsorbent to remove dyeing industry wastewater. Tea waste collection system and crushing treatment are the primary cost analysis. This cost could be compensated through dyeing industry wastewater treatment and tea waste disposal. Hence, large scale application of tea waste as low cost adsorbent is possible.

## 4. Conclusions

The kinetics process of MB adsorbed onto tea waste could be well described by a pseudo-second-order model. The kinetics process could be divided into a fast stage and a slow stage. The fast stage of adsorption could be finished within 5 min and removal rate was more than 90%. Langmuir could better fit the isotherm model and the maximum adsorption capacity is 113.1461 mg·g^−1^. The interaction between tea waste and MB referred to various mechanisms, including electrostatic interaction, hydrogen bond, ion exchange, π-π binding. Organic functional groups of tea waste played an important role during the MB adsorption. Therefore, tea waste could be acted as a potential adsorbent to remove dyeing wastewater.

## Figures and Tables

**Table 1:** Main functional groups of tea waste and MB loaded on tea waste.

Tea Waste			Assignment
Before Adsorption	After Adsorption	Difference	
3416	3406	+17	bonded –OH groups
2924	2925	0	aliphatic C–H group
2852	2852	0	aliphatic C–H group
1651	1644	+7	C=O stretching, Aromatic C=C, C=O/C=C stretching Amid Igroup
1530	1537	−7	secondary amine II group
1455	1455	0	C–H alkanes in aromatic rings
1371	1385	−14	C–H bending,–CH_3_ symmetric bending of CH_3_
1320	1331	−11	C–O stretching
1237	1244	−4	–SO_3_ stretching/P=O or COO vibration
1150	1150	0	C–O–C of polysaccharides
1036	1036	0	C–O–H stretching

**Table 2:** Relative proportion of different carbon types in tea waste.

Tea Waste	Chemical Shift (ppm), *δ*								
Relative proportion	0–46	46–65	65–90	90–108	108–145	145–160	160–185	185–225	225–250
(%)	22.61	12.90	23.76	10.46	14.21	5.71	9.96	0.13	0.26

Note: The spectra were integrated in the chemical shift (ppm) resonance intervals of 0–46 ppm (alkyl C, mainly CH_2_ and CH_3_ sp^3^ carbons), 46–65 ppm (methoxy and N alkyl C from OCH_3_, C-N and complex aliphatic carbons), 65–90 ppm (O-alkyl, such as alcohols and ethers), 90–108 ppm (anomeric carbons in carbohydrate-like structure), 108–145 ppm (aromatic and phenolic carbon), 145–160 ppm (oxygen aromatic carbon and olefinic sp^2^ carbons), 160–185 ppm (carboxyl, amides and ester) and 185–225 ppm (carboxyls).

**Table 3:** Adsorption kinetics and isotherm parameters of methylene blue on tea waste for various models.

**Pseudo-First-Order**			**Pseudo-Second-Order**			
*k*_1_ (L·min^−1^)	*q_e_* (mg·g^−1^)	R^2^	*k*_2_ (L·min^−1^)		*q_e_* (mg·g^−1^)	R^2^
39.726	23.323	0.9625	0.2317		24.077	0.9908
**TC models**			**Elovich**			
		R^2^	$\frac{1}{\beta }\ln(\alpha \beta )$		$\frac{1}{\beta }$	R^2^
F_fast_	F_slow_	0.9847	0.0071		11.9523	0.9159
0.7899	0.2101					
k_fast_	k_slow_					
852.8291	20.3117					
**Langmuir**			**Freundlich**			
*b* (L·mg^−1^)	*Q*_0_ (mg·g^−1^)	R^2^		*K_f_*	1/*n*	R^2^
0.08372	113.1461	0.9748		21.3460	0.3524	0.9159
**Temkin**			**D-R**			
*b_T_* (J·mol^−1^)	*K_T_* (L·mg^−1^)	R^2^	*q_m_* (mg·g^−1^)	*B* (mol^2^·kJ^−1^)	*E* (kJ·mol^−1^)	R^2^
103.466	0.7790	0.9727	124.1802	1.0330 × 10^−8^	6.956	0.9638

**Table 4:** Comparison of MB sorption capacity of tea waste with that of different sorbents-based on tea waste (*Q*_0_ obtained from Langmuir fitting).

Adsorbent	*Q*_0_ (mg·g^−1^)	pH	References
rejected tea (particle size in the range 250–355 μm)	147 (30 °C), 154 (40 °C), 156 (50 °C)	pH of 6–7	[11]
spent tea leaves (0.5–1.0 mm)	300.052	without changing the solution pH	[12]
tea waste (180–300 μm)	85.16	pH of 8	[13]
NaOH-modified rejected tea	242.11	pH of 7	[14]
Tea waste (less than 150 μm)	113.1461	pH unadjusted	This study

**Table 5:** The release of Ca^2+^, Mg^2+^, Na^+^, K^+^ during MB adsorption by tea waste at 35 °C.

Samples	The Net Amountof Released Cations (Mequivg^−1^)				Sum
	Ca^2+^	K^+^	Mg^+^	Na^+^	
100 mg·L^−1^–35 °C	0.02545	−0.00679	0.001938	0.073848	0.09444
100 mg·L^−1^–45 °C	0.0157188	−0.00955	0.002167	0.05962	0.067954
100 mg·L^−1^–45 °C	0.0186688	−0.01212	0.002333	0.055554	0.064441

Note: 100 mg·L^−1^–35 °C means the concentration of MB was 100 mg·L^−1^ and the operating temperature was 35 °C during the adsorption kinetics experiment.
